# Pediatric focal segmental glomerulosclerosis: favorable transplantation outcome with plasma exchange

**DOI:** 10.1186/s13052-021-01188-0

**Published:** 2021-12-14

**Authors:** Fatina I. Fadel, Hafez M. Bazaraa, Mohamed A. Abdel Mawla, Doaa M. Salah

**Affiliations:** 1 Department of Pediatrics, Pediatric Nephrology Unit, Kasr Al-Ainy Faculty of Medicine, Cairo University, Cairo, Egypt; 2 Department of Pediatrics, National Research Center, Cairo, Egypt

**Keywords:** Children, Focal segmental glomerulosclerosis (FSGS), Kidney transplantation, Perioperative plasma exchange, Recurrence

## Abstract

**Background:**

Although kidney transplantation (KTX) is the treatment of choice for pediatric end stage kidney disease (ESKD); concerns for recurrence in cases of focal segmental glomerulosclerosis (FSGS) are still present. This study aimed to investigate the outcome of KTX in children with ESKD secondary to FSGS, with implementation of preemptive perioperative plasma exchange (PE) for non-genetically proven patients.

**Methods:**

Forty FSGS pediatric kidney transplant recipients were studied. Of them: 12 patients (30%) had genetically proven NPHS2 mutations/familial and 28 (70%) were sporadic FSGS patients. All sporadic patients electively received 6 perioperative PE sessions. Patients with recurrence of proteinuria (*n* = 13; including 3 patients with genetic/familial and 10 patients with sporadic FSGS) were managed with PE and Rituximab (RTX). Kaplan-Meier curves were used to analyze graft and recurrence free survival data.

**Results:**

The mean follow-up duration after KTX was 3.8 ± 2.86 years. Recurrence of proteinuria was encountered early postoperative in 11 patients (27.5%) and late (1.6 and 2.9 years after KTX) in 2 patients (5%). All patients with early recurrence achieved complete remission, while patients with late recurrence developed graft failure. Current serum creatinine and proteinuria levels were not different in patients received PE (*n* = 31) and patients did not PE (*n* = 9) (*p* = 0.308 and 0.287 respectively). Current serum creatinine and proteinuria levels in sporadic patients (*n* = 28) after prophylactic perioperative PE were not different from those of genetic/ familial patients (*n* = 12) (*p* = 0.303 and 0.144 respectively). Proteinuria was less in patients underwent native nephrectomy than others immediately postoperative and at assessment (*p* = 0.002 & 0.0031 respectively). One-year graft and patient survival was 93.8% with a mean 1-year serum creatinine of 0.67 ± 0.25 mg/dl. Three graft losses (7.5%) were due to chronic rejection 3.3, 3.75 and 4.17 years after KTX and 2 patients’ mortality (5%) occurred early postoperative (first 2 weeks).

**Conclusion:**

FSGS transplanted children have favorable outcomes with perioperative PE for non-genetically proven cases. Early recurrence after KTX can be successfully managed with PE and RTX.

## Background

Focal segmental glomerulosclerosis (FSGS) is the third leading cause of chronic kidney disease (CKD) in children and accounts for 11.7% of end-stage kidney disease (ESKD) patients who undergo kidney transplantation (KTX) [1].

The incidence of recurrent FSGS after KTX has been a subject of immense research and intense discussion. The rate of recurrent FSGS varies from 20 to 50% after the first KTX and can reach up to 100% after subsequent transplants [2]. The risk of recurrence is inversely related to age and increases significantly in patients who progress rapidly to ESKD [3]. Additionally, recurrence is more frequent in patients with primary idiopathic FSGS than in those with the familial/genetic type [4].

Although idiopathic FSGS is strongly believed to be due to a circulating permeability factor (CPF), there is no accurate biomarker to predict recurrence [5]. Since recurrence of FSGS was historically considered a significant risk factor for graft loss in up to 50% of cases, it was considered a relative contraindication of KTX. Graft outcomes, however, in patients with recurrent FSGS continue to improve significantly [6].

The role of pre-emptive plasma exchange (PE) or immunoadsorption (IA) as a prophylactic strategy was established a long time ago with a variable success rate in patients with a high risk of FSGS recurrence [7]. However, there are no controlled trials to evaluate the efficacy of PE in children at risk for recurrent FSGS [8, 9]. To date, there have been no widely agreed upon guidelines for the treatment of recurrent FSGS after KTX, but treatment is based mainly on PE and rituximab (RTX) [10].

In this study we aimed to investigate the outcome of KTX in children with ESKD secondary to primary FSGS, with implementation of elective perioperative PE for non-genetically proven patients.

## Patients & methods

### Patients

**Fig. 1:**
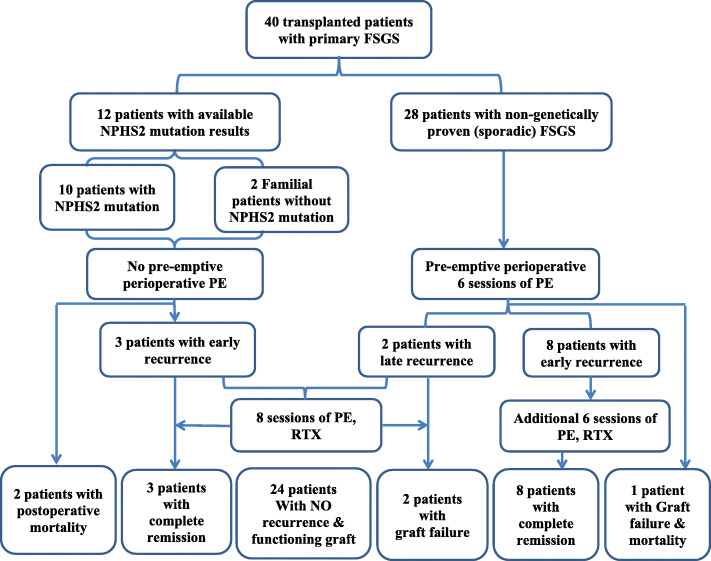
Flowchart describing FSGS type, patient management and their outcome

Forty pediatric kidney transplant recipients (KTRs) with ESKD due to primary FSGS received their first renal graft were included into the study. All included patients received living donor renal transplant and/ or followed up for at least 1 year at Pediatric Kidney Transplantation Unit, Cairo University Children Hospital (CUCH) between 2010 and 2020. Pre-transplantation results of genetic analysis for NPHS2 gene mutation were available in 12 patients only. Of them ten patients (25%) were proved to have NPHS2 gene mutation, and two patients (5%) with familial FSGS without NPHS2 gene mutation (Both were products of consanguineous parents; one with sibling mortality on dialysis due to FSGS & the other with ESKD sibling secondary to FSGS). Twenty eight (70%) patients had sporadic (non-genetically proven/ familial) FSGS **(**Fig. 1**)**. Transplanted children with ESKD due to secondary FSGS or those with missing data were excluded from the study.

## Method

This is a retrospective observational cohort study. The study was approved by the ethical committee of Pediatric Nephrology Unit, Pediatric Department, Cairo University. All procedures followed were in accordance with the Helsinki Declaration of 1964. Data were collected by reviewing of patients` records and by direct clinical/ laboratory evaluation of patients peri-operatively and during follow up visits.

Bilateral native nephrectomy was indicated in 13 (32.5%) patients based on their pre-transplantation heavy range of proteinuria (> 1000 mg/m^2^/day) & unilateral intraoperative native nephrectomy in 9 (22.5%) patients due to their sub-nephrotic range of proteinuria (100-1000 mg/m^2^/day). Eighteen patients (45%) were anureic at time of KTX and did not undergo native nephrectomy. The reason for native nephrectomy was to control nephrotic syndrome and to avoid confusion about the source of proteinuria (such in case) after transplantation; whether from native kidneys or the renal graft.

KTX was performed preemptively (before initiation of regular dialysis) in 12 patients (30%) or KT after a period of dialysis in 28 patients (70%). Time option of KTX was determined individually based on donor availability, patient medical fitness for the operation and family preference/ related circumstances.

KTX related encountered complications were mainly in the form of; a) surgery related morbidities that necessitated reoperation included urinary leak (in 2 patients) and bleeding (in 1 patient), b) Sever infection in the form of early fulminant pneumonia and sepsis necessitating PICU admission (in 4 patients), c) Infections encountered during follow up periods necessitating hospital admission in the form of repeated urinary tract infection (in 2 patients), gastroenteritis (2 patients) and CMV infection (2 patient), d) Early (first 3 months) and delayed acute rejection was encountered in 5 (12.5%) and 8 (20%) patients respectively (total 14%/ patient-year).

All non-genetically proven /non familial idiopathic FSGS patients (Sporadic FSGS; *n* = 28; 70%) preemptively received six sessions of PE (three sessions preoperative starting day − 5 and three sessions during the first postoperative week) regardless their proteinuria state perioperative. For genetic / familial FSGS patients (*n* = 12; 30%) no preemptive preoperative PE sessions were performed due to their reported low risk of recurrence, however, surveillance of postoperative proteinuria in this group revealed recurrence in 3 patients for whom 8 sessions of postoperative PE were performed for therapeutic purpose. Recurrence of proteinuria (in *n* = 13 including 10 patients with sporadic FSGS and 3 patients with genetic/ familial FSGS) was managed with further 6 sessions of PE in sporadic FSGS patients & 8 sessions in genetic FSGS followed by 2–4 doses of weekly RTX (365 mg/m^2^/dose) in both types of FSGS patients.

Each PE session was performed using 1.5 plasma volume exchange with salt free albumin using Prismaflix Machine and were scheduled to be day after the other.

All patients (*n* = 40) were monitored for proteinuria according to KDIGO guidelines of proteinuria screening for post-transplant idiopathic FSGS as following: daily testing for 1 week, weekly testing for 4 weeks, and every 3 months thereafter [11]. Protocol graft biopsy was performed 1 month postoperative for 25 patients (62.5%) only as protocol biopsies started to be implemented at our center since 2015 [12]. Indication biopsy was performed for patients upon their medical indication during their follow up visits. Pathological findings were reported according to Banff criteria current at the time of biopsy interpretation.

### Definitions

Recurrence of proteinuria was defined as urinary protein> 0.5 g/day. Early recurrence was defined as recurrence of proteinuria within the first 2 weeks after the operation while late recurrence was defined as recurrence of proteinuria after 1 year of KTX. Remission after treatment was defined as proteinuria < 0.5 g/day (complete remission) or proteinuria between 0.5–3.5 g/day (partial remission) [8]. Graft survival was defined as functioning graft with no need for re-establishment of regular dialysis while recurrence free survival was defined as functioning graft with no recurrence of proteinuria after KTX.

### Statistical analysis

Nominal data were expressed as frequencies and percentage, parametric data as means and standard deviations and non-parametric data as median and interquartile range (IQR). Two group comparisons were done using Chi-square test for qualitative data. Two independent group comparisons with quantitative data and parametric distribution were done by using independent t-test. *P* value < 0.05 was considered significant. Kaplan-Meier curves were used to present graft and recurrence free survival.

## Results

The study included 40 patients (20 males & 20 females), with mean age of 10.5 ± 4.03 years. Their mean KTX age was 9.21 ± 3.88 years with mean post-transplant follow-up duration of 45.6 ± 34.35 months. Thirteen patients (32.5%) are products of consanguineous marriage with 10 patients (25%) have sibling affection with FSGS (familial FSGS). Demographic, clinical & transplantation related data of the studied patients are displayed in Table 1**.**

**Table 1:** Demographic, clinical & transplantation data of the study group (*n* = 40)

	Mean	SD
Follow up duration before TX (mo)	33.54	±27.81
Dialysis duration (years)	3.41	±1.958
Pre-TX proteinuria (mcg/d)	3017	±1425
TX weight (kg)	20.14	±6.214
Cold ischemia time (min)	66.32	±16.93
Post-operative hospital stay (days)	16.64	±12.72
Immediate post-TX proteinuria (mcg/d)	2895	±954
*Current weight (kg)	35.44	±18.91
*Current height (cm)	127.2	±35.34
Serum creatinine after 1 yr (mg/dl)	0.668	±0.251
Serum creatinine after 3 yr (mg/dl)	0.728	±0.145
Post-TX GFR	94.92	±22.4
Current serum creatinine (mg/dl)	0.728	±0.145
Current GFR	65.88	±35.16
Current protein in urine (mcg/d)	27.9	±51.66

TX (transplantation), GFR (glomerular filtration rate)

*Weight and height at assessment after follow-up duration of 45.6 ± 34.35 months

Twenty patients (50%) received their renal grafts from their mothers, eleven (28%) from their father & others (9 patients; 22%) received renal grafts from non-genetically related donor. The mean donor age at transplantation was 39.16 ± 10.587 years.

For non-genetic FSGS patients (*n* = 28); immunosuppression therapy consisted of antibody induction with ATG and triple therapy classic protocol that consists of steroids, CNI and mycofenolate. CNI was in the form of cyclosporine (CsA) 10 and tacrolimus in 18 patients. For genetic/ familial FSGS patients (*n* = 12); immunosuppression therapy consisted of antibody induction (ATG in 7 patients and with basilximab in 5 patients) and triple classic therapy protocol. CNI was in the form of CsA in 6 patients and tacrolimus in 6 patients. The mean steroid dose for the whole FSGS cohort at assessment was 5.8 ± 3.2 mg/day (ranging between 1.25 & 10 mg/day).

Early graft function was excellent in 34 patients (85%), delayed (DFG) in 4 patients (10%) & transient oliguria due to acute tubular necrosis was encountered in 2 patients (5%).

By comparing transplant recipients with genetic/familial FSGS & those with non-genetic (sporadic; subjected to pre-emptive PE) FSGS **(**Table 2**),** we found that; immediate (day 1) post-transplantation proteinuria was significantly more in sporadic than genetic FSGS patients (2514.65 ± 915 versus 546.25 ± 100.1mcg/day; *p* = 0.00001). After 1 week post-operative (after at least 3 sessions of PE for sporadic FSGS patients), proteinuria significantly decreased in sporadic FSGS recipients (2514.65 ± 915 versus 954 ± 321 mcg/day; *p* ≤ 0.001). Graft function (serum creatinine) at assessment was not significantly different between patients with sporadic FSGS and those with genetic/familial FSGS (0.754 ± 0.3 versus 0.605 ± 0.3 mg/dl; *p* = 0.1437).

**Table 2:** Comparison between genetic & sporadic FSGS patients

	Sporadic FSGS (*n* = 28)	Genetic/Familial (*n* = 12)	*P*-value
Pre-TX proteinuria (mcg/d)	3003 ± 1505	2101 ± 608	0.0388
D1 Post-TX proteinuria (mcg/d)	2514.654 ± 915	546.254 ± 100.1	0.00001
D7 Post-TX proteinuria (mcg/d)	954 ± 321	189 ± 56	0.00001
Current Proteinuria (mcg/d)	35.29 ± 60.94	10.62 ± 7.53	0.3027
Current serum creatnine (mg/dl)	0.754 ± 0.3	0.605 ± 0.3	0.1437

TX (transplantation)

Recurrence of proteinuria was encountered in 13 patients (32.5%) out of the whole studied FSGS cohort. Of them; 2 patients were genetically proven NPHS1 mutation and 1 patient with NPHS2 mutation negative familial FSGS. All patients with recurrence developed early proteinuria (first 15 days postoperative) except for 2 sporadic FSGS patients; in whom proteinuria appeared 21 and 35 months after KTX and was associated with chronic active antibody mediated rejection (ABMR). All patients with early recurrence of proteinuria (*n* = 11) achieved complete remission after therapeutic PE and RTX. Patients with delayed proteinuria (*n* = 2) were not responsive to therapy and developed progressive proteinuria, graft failure (re-establishment of regular dialysis) 3.75 and 4.17 years after KTX**.** Three patient’s mortalities (7.5%) were reported; 2 genetic FSGS patients with early postoperative mortality and one sporadic FSGS patients with graft failure due to chronic ABMR and mortality on regular dialysis **(**Fig. 1**)**.

Table 3 illustrates that patients received PE (*n* = 31; including 28 risky sporadic patients and 3 genetic/ familial patients experienced early recurrence) have similar graft outcome to that of the reported prognostically better genetic/familial patients who did not experience recurrence and did not receive PE (*n* = 9). Current serum creatinine and urine protein levels were not significantly different between both group of patients (*p* = 0.308 and 0.287 respectively), signifying the improved outcome of sporadic cases and cases with recurrence after PE to approach that of genetic cases with reported better prognosis.

**Table 3:** Comparison between patients received and those who did not receive PE as regard current serum creatinine and proteinuria level

	None-PE patients (*n* = 9)	PE patients (*n* = 31)	P-value
Current serum creatnine (mg/dl)	0.812 ± 0.432	0.701 ± 0.23	0.3079
Current urine protein (mcg)	29 ± 10.11	33 ± 9.7	0.2872

To evaluate impact of native nephrectomy on post-transplantation proteinuria; we compared patients underwent pre-transplant native nephrectomy (*n* = 22) & those who did not have medical indication for nephrectomy (*n* = 18) **(**Table 4**).** Both immediate post-transplantation as well as current (after mean follow up duration of 3.8 years) proteinuria were significantly elevated in non-nephrectomized than nephrectomized patients (*p* = 0.002 & 0.0031 respectively).

**Table 4:** Comparison between patients with & without native nephrectomy(s)

	Nephrectomized patients (*n* = 22)	Non nephrectomized patients (*n* = 8)	*P*-value
Current Creatnine	0.854 ± 0.334	0.798 ± 0.356	0.6115
Post-TX proteinuria (mcg/d)	2050 ± 805	3101 ± 1102.56	0.002
Current proteinuria (mcg/d)	27 ± 10.5	39 ± 14	0.0031

TX (transplantation)

**Fig. 2:**
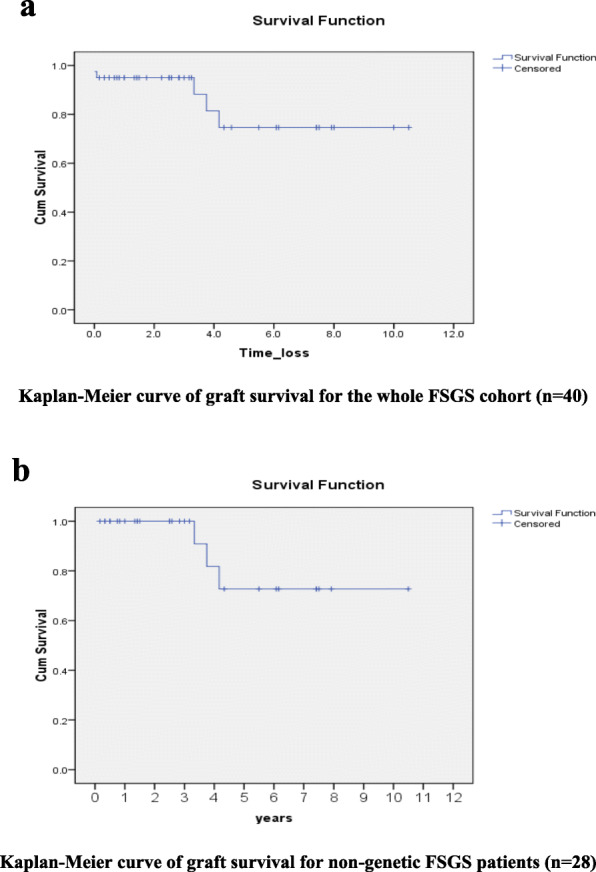
**a** Kaplan-Meier curve of graft survival for the whole FSGS cohort (*n* = 40). **2b** Kaplan-Meier curve of graft survival for non-genetic FSGS patients (*n* = 28)

**Fig. 3:**
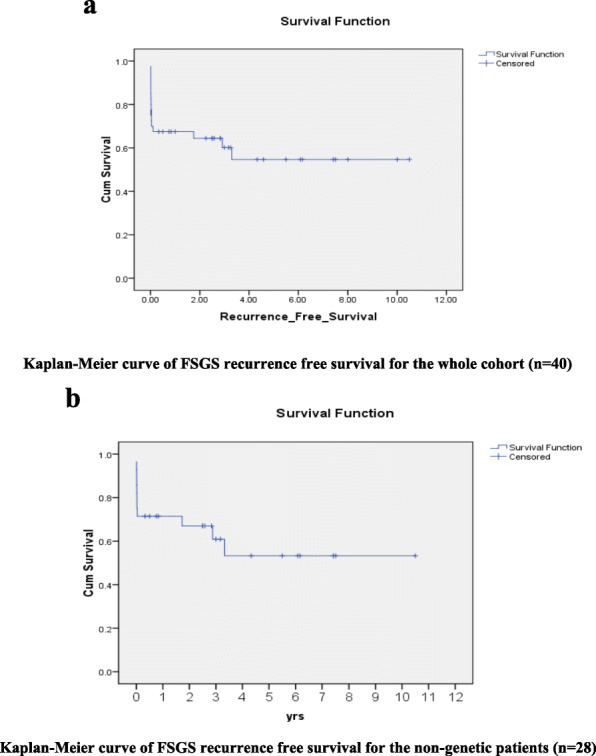
**a** Kaplan-Meier curve of FSGS recurrence free survival for the whole cohort (*n* = 40). **3b** Kaplan-Meier curve of FSGS recurrence free survival for the non-genetic patients (*n* = 28)

One year graft and patient survival of the whole cohort was 93.8% with a mean (±SD) one year serum creatinine of 0.67 ± 0.25 mg/dl. Three graft losses (7.5%) were due to chronic active ABMR 3.3, 3.75 and 4.17 years after KTX in addition to two postoperative mortalities were reported. Kaplan-Meier curves in Fig. 2a and b illustrate overall graft survival data of the whole FSGS cohort (*n* = 40) and that of non-genetic FSGS patients (*n* = 28) respectively. Figure. 3a and b illustrate recurrence free survival data of the whole FSGS cohort and that of non-genetic FSGS patients respectively.

## Discussion

FSGS has been reported as having poorer transplant outcomes in children than most other causes of ESKD largely because of disease recurrence [13]. Proteinuria may herald recurrent FSGS even if an early biopsy does not show glomerular abnormalities [14]. Absence of a causative mutation represents the major risk factor for FSGS recurrence, while transplant can be curative in genetic forms of the disease [15]. Since recurrent FSGS was postulated to be caused by CPF affecting podocyte structure and function, interventions such as PE and RTX may result in improved outcomes in treatment rather than prevention of recurrence [16].

We already have reported our 10 year experience of KTX in children as of 2018 as a specialized Center in Egypt [12]. In this study we displayed the role of pre-emptive PE in decreasing proteinuria and improving outcome of FSGS after a mean of 3.8 years follow up after KTX.

This study represents a retrospective analysis of a cohort of 40 pediatric KTRs with ESKD caused by primary FSGS. Non-genetically proven/ familial recipients (28 patients; 70%) received perioperative pre-emptive six PE sessions. Eleven patients; 27.5% (including 3 genetic/familial FSGS) developed early recurrence were successfully treated by PE/RTX, while 2 patients (5%) with late proteinuria developed graft failure.

Our overall incidence of disease recurrence after KTX among non-genetic patients (10/28; 35.7%) is less than what was reported by Morello & his co-workers [15] in their Italian experiences (53%). Additionally; previous data in the literature, stretching back almost three decades, reported higher incidence of recurrence than our report [13, 17, 18].

We reported significant reduction in early proteinuria of non-genetic FSGS transplant recipients with perioperative PE sessions [D1 to D7 (2514.65 ± 915 versus 954 ± 321mcg/day; *p* ≤ 0.001) and D7 to time of assessment after mean 3.8 years follow up duration (954 ± 32 versus 35.29 ± 60.94 mcg/day; p ≤ 0.001).

Bouts & his colleagues performed survey among European Society of Pediatric Nephrology (ESPN) members that gives insight into the variation in policies regarding the prevention and treatment of FSGS recurrence with response rate of 15% (59/391 active members), mostly all by pediatric nephrologists. In this survey, one-third of the respondents treated pre-emptively with PE and/or RTX or CsA [18].

To date, PE is of uncertain value in primary FSGS in the native kidney [19] unlike CsA which is the only evidence-based treatment for FSGS before KTX [18, 20]. In the transplant kidney, pre-emptive PE implementation in FSGS patient with anticipated high recurrence risk makes sense: remove the injurious; podocyte toxic CPF from the blood before causing irreversible structural damage. However, a prospective, randomized trial of apheresis therapy versus placebo has never been conducted for either native kidney or post-transplant FSGS.

In the present study we reported 1 year graft and patient survival of the whole cohort of 93.8%, with a mean 1 year graft function (serum creatinine) of 0.67 ± 0.25 mg/dl. Moreover; we analyzed graft survival and recurrence free survival data using Kaplan Mayer curves (Fig. 3a and b respectively) with very promising results.

The effect of PE on long-term graft survival is especially unknown [19]. However, reported response rates were 50–90% higher than expected with other approaches (30–40%) making it a reasonable approach until more data become available [8].

Genetic FSGS patients have defective components of the kidneys, rather than circulating factors and therefore their risk of recurrence is low [4, 21]. However apart from disease recurrence, which mostly occurs early after KTX, graft outcome differences between genetic and non-genetic FGSG patients have not yet been discussed. Interestingly; we reported no difference in graft function in term of serum creatinine in sporadic than genetic FSGS transplant recipients (*p* = 0.1437) after a mean follow up duration of 3.8 years with implementation of pre-emptive PE to sporadic patients. This means that the postulated worse outcome of sporadic than genetic FSGS patients after KTX that is associated with increased risk of recurrence can be partially overcomed by pre-emptive PE implementation.

Native kidney nephrectomy prior to KT has been suggested by some as a preventive measure of recurrence [22], but it has not been effective and has even shown a higher risk of recurrence in other reports [23, 24].

Native nephrectomy was specifically analyzed among our cohort as one of the potentially modifiable risk factors of proteinuria recurrence after KTX. We reported positive impact of native nephrectomy on the graft in term of less prost-transplant proteinuria (both early and current; *p* = 0.0296 & 0.0441 respectively). Our finding regard native nephrectomy does not go with what was recently published by Uffing & his co-workers [25]. They reported prior nephrectomy as a significant risk for recurrence, but their number of participants is too small to draw definitive conclusions. One of the hypotheses is that the native kidneys left in situ may act as a “sponge” to absorb the potential pathogenic CPF, leading to reduction of the free CPF that may injure the transplanted kidney [26]. Our explanation, however, of less proteinuria in patients with native nephrectomy is based on the absence of proteinuria derived from the native kidneys after KTX. This contradictory findings could be simply explained by the fact that severe nephrosis from FSGS, which may also have conferred a higher risk for recurrence, might have been the indication for nephrectomy [19].

This study has a number of limitations. First; the retrospective nature of the study and being a cohort study with lack of controls of sporadic FSGS patients who did not receive PE for comparison of outcomes. Second; lack of genetic testing of the whole cohort and the limitation of genetic testing to only NPHS2 mutation for non-genetically proven familial patients. Third; lack of pathological analysis of most of patients with early proteinuria. Fourth; other confounders that impact outcome (as KTX timing, donor/recipient related morbidities and using serum creatinine for graft function assessment) or causing proteinuria as chronic rejection (particularly in late proteinuria) were not analyzed in details in this study. Further studies to overcome these limitations and to investigate the potentially modifiable predictors of outcome in this particular group of transplant recipients are highly recommended.

**In conclusion;** FSGS transplanted children have favorable outcomes with implementation of pre-emptive perioperative PE for non-genetically proven cases. Early recurrence can be successfully managed with PE and RTX. Native nephrectomy in FSGS is still questionable however; it omits post-transplant proteinuria originating from native kidney that may confuse the physician with recurrence after KTX.
